# Evaluation of the Prevention and Control Efficacy of Different Immunization Regimens Against Porcine Epidemic Diarrhea Virus

**DOI:** 10.1155/tbed/3164025

**Published:** 2026-07-20

**Authors:** Chen Sun, Juan Jin, Li Zhao, Meng Hao, Maojie Lv, Endong Bao

**Affiliations:** ^1^ Pathology Laboratory, Nanjing Agricultural University, Nanjing, Jiangsu, China, njau.edu.cn; ^2^ Ringpu Biotechnology Co., Ltd., Tianjin, China; ^3^ Jiangsu Synthgene Biotechnology Co., Ltd., Nanjing, Jiangsu, China

**Keywords:** immunization regimen, passive immune protection, *PEDV*, vaccine platform

## Abstract

Porcine epidemic diarrhea virus (*PEDV*) is an infectious pathogen that induces severe injury to the small intestinal epithelium of pigs. Pigs of all ages are susceptible to infection, which typically presents with watery diarrhea as the main clinical manifestation. In neonatal piglets, *PEDV* infection results in nearly 100% morbidity and mortality rates exceeding 80%. Therefore, selecting an appropriate immunization regimen for pregnant sows is particularly critical, as effective maternal vaccination enables the transfer of robust passive immunity to neonatal piglets, thereby protecting them against *PEDV* infection. In this study, three vaccination regimens (inactivated vaccine [IV] + IV, attenuated live vaccine [AV] + IV, and mRNA vaccine [MV] + MV) were used to immunize pregnant sows, and the passive immune protection conferred to neonatal piglets was subsequently evaluated. The results showed that piglets in the control group failed to acquire passive immune protection, with all piglets (5/5) developing diarrhea after challenge. The AV + IV regimen induced strong cellular immune responses and moderate humoral immunity, resulting in complete passive protection of piglets (5/5) following challenge, with only low levels of *PEDV* RNA detected in rectal swabs, which persisted for no more than 7 days. The IV + IV regimen elicited optimal humoral immune response but the weakest cellular immunity, whereas the MV + MV regimen induced moderate levels of both humoral and cellular immunity; piglets in both groups achieved partial passive protection (4/5), and relatively higher *PEDV* RNA loads, although still lower than those observed in the control group, were persistently detected in feces for up to 14 days. Collectively, the comparative analysis of passive immune protection conferred by the three immunization regimens indicated that the AV + IV strategy achieved superior protective efficacy, yielding superior outcomes compared with the MV + MV and IV + IV regimens. As MVs represent a novel and rapidly evolving platform for veterinary biologics, further technological innovation and systematic optimization are likely to enhance their immunogenicity and corresponding protective potency in subsequent studies.

## 1. Introduction

Porcine epidemic diarrhea (PED) is an acute infectious disease caused by the PED virus (*PEDV*), which infects villous enterocytes in the small intestine [1]. Clinical manifestations of *PEDV* infection include diarrhea, vomiting, and dehydration, while neonatal piglets additionally suffer from high mortality rates [2]. Since its initial identification, *PEDV* has caused substantial economic losses to the global swine industry, and PED remains a major contributor to reduced piglet survival globally [3, 4]. The most effective strategy for protecting neonatal piglets against *PEDV* infection is the acquisition of sufficient maternally derived antibodies through colostrum and milk [5]. However, numerous studies have reported suboptimal immune efficacy of both inactivated and attenuated *PEDV* vaccines [6], which has been attributed to inappropriate immunization regimens and continuous viral strain mutations [7], leading to antigenic mismatch. *PEDV* is classified into two major genotypes, GI and GII, with the *spike* (*S*) gene of GII strains exhibiting characteristic insertion and deletion mutations relative to those of GI strains. These genetic variations markedly reduce viral immunogenicity and cross‐protective efficacy [8], underscoring the importance of selecting vaccines that match the currently circulating *PEDV* genotypes. *PEDV*‐inactivated vaccines (IVs) exhibit favorable safety profiles. However, viral immunogenicity is partially compromised during the inactivation process, often requiring booster immunizations [9] to induce humoral immune responses [10]. In contrast, immunization with attenuated live vaccines (AV) elicits robust cellular immune responses by activating lymphocytes, promoting the differentiation of CD4^+^ and CD8^+^ T cells [11, 12], and enhancing the secretion of cytokines such as IL‐1α and IFN‐γ [13]. Serial passage of *PEDV* in Vero cells for more than 120 generations effectively attenuates viral virulence while preserving immunogenicity [14], although the potential risk of virulence reversion remains [15, 16]. In recent years, the development of novel vaccine platforms, including mRNA vaccines (MVs), has provided alternative strategies for PED prevention [17]. The *S* protein represents the most promising antigenic target for MV development [1, 17–19] as it exhibits high genetic variability and plays a central role in viral infection, inducing neutralizing antibodies and modulating in vivo viral virulence [20]. Accordingly, the *S* gene is considered the primary virulence‐associated gene [6]. Intestinal antibody‐secreting cells (ASCs) are critical for resistance to enteric viral infections [21], and secreted IgA is strongly correlated with neutralizing antibody titers [22]. Moreover, the synergistic action of IgG and IgA further enhances virus‐neutralizing activity [23]. Taken together, in the context of the persistent global threat posed by *PEDV*, matching vaccines to the circulating genotype and implementing rational immunization strategies are essential for improving vaccine immunogenicity and protective efficacy.

In this study, three *PEDV* vaccines, an IV, an AV, and an MV, were developed based on the *PEDV* JX strain. Pregnant sows were immunized with three distinct regimens: IV + IV, AV + IV, and MV + MV. The passive immune protection conferred to neonatal piglets was subsequently evaluated following challenge with a homologous *PEDV* strain. The results demonstrated that the AV + IV regimen provided the most effective passive immune protection, outperforming both the MV + MV and IV + IV regimens. Although further optimization is required, MVs have demonstrated considerable potential in antiviral applications [24–26] and represent a promising platform for future vaccine development.

## 2. Materials and Methods

### 2.1. Vaccine Preparation

An AV: The *PEDV* JX strain was serially passaged in Vero cells to achieve attenuation at Passage 240. The live *PEDV* antigen was obtained via expanded culture, clarification, concentration, and purification, followed by lyophilization to prepare the attenuated *PEDV* vaccine (JX 240 strain; designation: AV). The vaccine contained 10^6.0^ TCID_50_/mL viable antigen (Batch Number H20240406).

IV: The *PEDV* JX strain was propagated in Vero cells, and the viral antigen was harvested through clarification, concentration, purification, and inactivation. The inactivated antigen was emulsified with ISA 201 adjuvant (SEPPIC, France) to produce the *PEDV*‐IV (JX strain; designation: IV), which contained 10^7.0^ TCID_50_/mL of inactivated antigen (Batch Number M20240402).

MV: The *PEDV* JX strain *S* gene was cloned into the pVAX1 vector to construct the recombinant plasmid. The plasmid was propagated in Stbl3‐competent cells via fermentation, followed by linearization and transcription to synthesize mRNA. The mRNA was encapsulated in lipid nanoparticles (LNPs) to prepare the *PEDV* MV (JX strain; designation: MV), with a concentration of 100 μg/mL mRNA (Batch Number PED2024019).

All IV, AV, and MV employed in the present trial were manufactured and supplied specifically for clinical use by Ringpu Biotech Co., Ltd. and Jiangsu SynthGene Biotechnology Co., Ltd.

### 2.2. Virus for Detection

*PEDV* JX strain (GenBank: PZ376861): A genotype GⅡb strain, serially passaged in Vero cells to the F5 generation. Seven‐day‐old piglets orally challenged with 3.0 mL viral suspension containing 10^3.0^ TCID_50_/mL all developed diarrhea, with 100% mortality observed. This strain was isolated and characterized by Ringpu Biotech Co., Ltd.

*PEDV* HBXT strain (GenBank: MH816969.1): A genotype GⅡa strain, isolated and identified by the Lanzhou Veterinary Research Institute, Chinese Academy of Agricultural Sciences.

*PEDV* TJ strain (GenBank: PZ376862): A genotype GI strain, isolated and identified by Ringpu Biotech Co., Ltd.

### 2.3. Vaccine Immunization and PEDV Challenge

Vaccines were administered strictly in accordance with the manufacturer’s instructions. Twelve healthy and susceptible sows (double‐negative for *PEDV* antigen and antibody) at 30 days of gestation were selected and randomly divided into four experimental groups (*n* = 3 sows per group). Primary and booster immunizations were administered via cervical intramuscular injection at 30 and 16 days prior to farrowing, respectively. The immunization regimens for Groups 1–4 were as follows: IV + IV, AV + IV, MV + MV, and phosphate‐buffered saline (PBS, 0.01 M, pH 7.2) + PBS, with a dosage of 2.0 mL per sow. Sows in each group were housed in individual isolation pens under identical environmental conditions.

After farrowing, 12 healthy piglets were randomly selected from each immunization group. Of these, five piglets were subjected to continuous blood collection for antibody detection, while the other seven were transferred to the laboratory and challenged with *PEDV* at 7 days of age. Each piglet was orally administered 3.0 mL of *PEDV* JX strain viral suspension (viral titer: 10^3.0^ TCID_50_/mL). Among the challenged piglets, two individuals were necropsied at 5 days postchallenge (dpc), and the remaining five were necropsied at 14 dpc. In addition, two age‐matched piglets were set up as the blank control group. Piglets were euthanized strictly in accordance with animal experimental ethical guidelines. In brief, a 20% sterile sodium pentobarbital solution was injected into the anterior vena cava at a dose of 150 mg/kg body weight. Euthanasia was confirmed by the disappearance of the corneal reflex and spontaneous breathing. After euthanasia, necropsy was performed immediately. All animal carcasses and experimental waste were disposed of by autoclave sterilization in compliance with institutional biosafety guidelines.

### 2.4. Neutralizing Antibody Titer Detection

Blood samples were collected from sows at 14, 30 (day of farrowing), 60, 90, and 120 days post‐primary immunization (DPP). On the day of farrowing, both blood and colostrum were collected from the sows. In addition, five healthy piglets born to immunized sows were randomly selected from each group, and blood samples were collected at 7, 14, 21, 28, 35, and 42 days of age for neutralizing antibody detection. Serum samples were inactivated at 56°C for 30 min. Milk samples were subjected to two freeze–thaw cycles and centrifuged at 3000 × *g*, and the supernatant was collected. The samples were serially diluted by twofold in DMEM (Gibco, USA). Each dilution was mixed with an equal volume of *PEDV* suspension (viral titer: 200 TCID_50_/0.1 mL) and incubated at 37°C for 1 h. A 0.1 mL aliquot of the mixture was inoculated onto 96‐well plates containing confluent Vero cell monolayers (cell culture medium was discarded and plates were washed twice with DMEM). DMEM medium supplemented with 20 μg/mL trypsin was added at 0.1 mL per well, with four replicate wells per dilution. Plates were incubated at 37°C for 72–96 h, and cytopathic effects (CPEs) were observed daily. The neutralizing antibody titer was defined as the highest dilution that protected more than 50% of the cells from CPE.

### 2.5. ELISA for PEDV S Protein‐specific Antibodies

*PEDV* S protein‐specific IgA and IgG antibodies in serum and milk samples were detected using an ELISA kit (Hongsheng Biotechnology Co., Ltd., China) following the manufacturer’s instructions. The samples were diluted 100‐fold, followed by incubation at 37°C for 1 h. After washing with wash buffer, the secondary antibody was added and incubated at 37°C for 30 min. Following additional washing, a chromogenic substrate was added for color development. The optical density (OD) value at 650 nm was measured using a microplate reader. The S/P ratio was calculated as follows: S/*P* = (sample OD value − mean OD value of negative controls)/(mean OD value of positive controls − mean OD value of negative controls). Samples were considered antibody‐positive if the S/P ratio was ≥0.2 (for IgA) or ≥0.4 (for IgG).

### 2.6. Flow Cytometry Analysis

Anticoagulated whole blood was collected from sows on the farrowing day. Porcine peripheral blood lymphocytes (PBLs) were isolated using a porcine lymphocyte separation medium (Dakewe Biotech Co., Ltd., China). Isolated lymphocytes were resuspended in flow cytometry buffer at a concentration of 1 × 10^6^ cells/200 μL per tube. Cells were stained with SPRD–conjugated mouse anti‐porcine CD3, FITC‐conjugated mouse anti‐porcine CD4, and PE‐conjugated mouse anti‐porcine CD8 monoclonal antibodies (Biolead, China) in the dark for 30 min. After centrifugation to discard the supernatant, the cells were washed twice with PBS. A BD flow cytometer was used to identify CD3^+^ CD4^+^ T cells and CD3^+^ CD8^+^ T cells, and their proportions relative to total T cells were calculated.

### 2.7. Quantification of IL‐4 and IFN‐γ in PEDV S Protein‐Stimulated Lymphocytes

Lymphocytes were isolated from the blood samples collected on the day of farrowing, stimulated with *PEDV* S‐protein, and then used for cytokine detection. 100 μL cell suspension (1 × 10^5^ cells) was added to each well of a 96‐well cell culture plate, followed by stimulation with 5 μg/100 μL eukaryotic recombinant *PEDV* S protein (Yinduo Biotechnology Co., Ltd., China) for 24 h. Culture supernatants were collected, and the concentrations of IL‐4 and IFN‐γ were quantified using commercial ELISA kits (Meimian, China) according to the manufacturer’s protocols.

### 2.8. Fecal Consistency Scoring Standard

Fecal consistency was scored daily postviral challenge to assess diarrhea using a 4‐point scale: 0, normal solid feces; 1 point, unformed solid feces; 2 points, semiliquid feces with solid components; and 3 points, watery feces without solid components. Piglets with a fecal score of more than 2 points were defined as having diarrhea.

### 2.9. Histopathological and Immunohistochemistry (IHC) Examination

At 5 dpc, two piglets from each group were euthanized for necropsy, and gross lesions were recorded. Jejunal tissue samples were collected and fixed in 10% neutral buffered formalin. Fixed tissues were processed through dehydration, clearing, paraffin infiltration, and embedding to prepare paraffin blocks. Serial 5 μm‐thick paraffin sections were prepared via sectioning, spreading, mounting, and baking. After dewaxing and rehydration, sections were subjected to hematoxylin–eosin (H&E) staining and IHC analysis. H&E staining: Hematoxylin stained the cell nuclei blue, while eosin stained the cytoplasm pink, enabling visualization of tissue microstructure and cellular morphology (e.g., villus integrity, epithelial damage, and inflammatory cell infiltration). IHC analysis: A mouse anti‐*PEDV* N protein monoclonal antibody (MyBioSource, USA) was used as the primary antibody, and a goat anti‐mouse IgG secondary antibody (Abcam, UK) was used for detection. IHC was performed to evaluate *PEDV* invasion of intestinal tissues, with positive signals indicating viral antigen localization.

### 2.10. Quantitative Real‐Time PCR (qRT‐PCR) for PEDV

After challenge, the rectal swabs were collected daily for qRT‐PCR. Fecal samples (1.0 g) were homogenized in 1.0 mL of sterile water for injection. A 200 μL aliquot of the homogenate was used for total RNA extraction. Primers and a TaqMan probe were designed targeting the *PEDV N* gene, with the following sequences. Forward primer: 5′‐GTCTGAAAAGCCAATCATTC‐3′; reverse primer: 5′‐TTGCCTCTGTTGTTACTC‐3′; probe: 5′‐CTGTTGTTGCCATTGCCACGA‐3′. qRT‐PCR was performed according to the TaqMan kit manufacturer’s instructions.

### 2.11. Data Analysis

All data were statistically analyzed and graphically visualized using GraphPad Prism 8 software (GraphPad Software, Inc., USA). Quantitative data are presented as the mean ± standard deviation (SD). Differences between the two groups were compared using an unpaired Student’s *t*‐test. For comparisons among multiple groups, one‐way analysis of variance (ANOVA) was performed, followed by a post hoc test (e.g., Tukey’s or Duncan’s multiple range test) to identify pairwise differences. Statistical significance was defined as a two‐tailed *p*‐value <0.05, with the following notation: “ ^∗^” indicates *p* < 0.05, “ ^∗∗^” indicates *p* < 0.01, and “ns” indicates no statistical significance.

## 3. Results

### 3.1. Immunization Regimens for Pregnant Sows and Dynamic Changes in Neutralizing Antibody Titers

**Figure 1:**
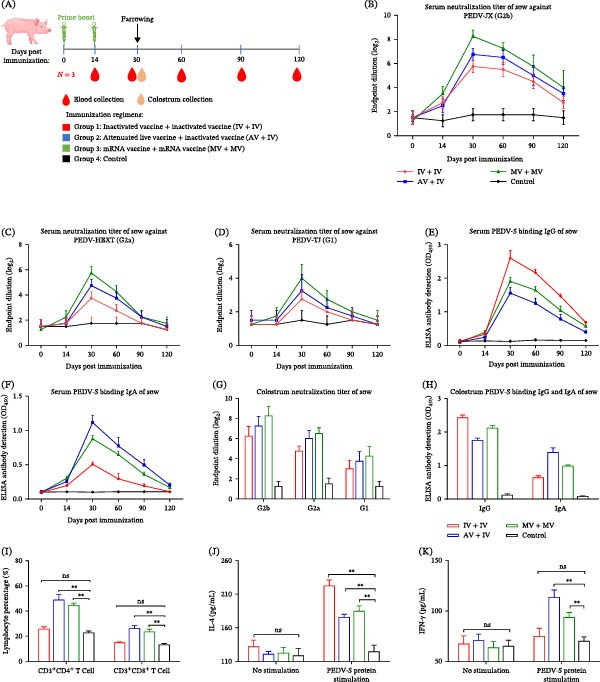
Immune antibody responses and T lymphocyte–specific reactions in pregnant sows following different immunization regimens. (A) Schematic of the immunization schedule for pregnant sows. Sows were immunized twice via intramuscular injection at 30 and 16 days prefarrowing. Four prime‐boost regimens were applied to Groups 1–4. Group 1 (IV + IV), inactivated vaccine + inactivated vaccine; Group 2 (AV + IV), attenuated live vaccine + inactivated vaccine; Group 3 (MV + MV), mRNA vaccine + mRNA vaccine; Group 4 (negative control), PBS + PBS (0.01 M, pH 7.2). (B–F) Kinetics of humoral immune responses. Blood samples were harvested from sows at 14, 30 (farrowing day), 60, 90, and 120 days post‐primary immunization to detect. (B) Neutralizing antibody titers against *PEDV* GⅡb; (C) cross‐neutralizing antibody titers against *PEDV* GⅡa genotype strain; (D) cross‐neutralizing antibody titers against *PEDV* GⅠ genotype strain; (E) *PEDV* S protein‐specific IgG levels measured via ELISA; (F) *PEDV* S protein‐specific IgA levels measured via ELISA. (G, H) Antibody concentrations in colostrum. Colostrum was collected at farrowing to quantify (G) neutralizing antibody titers and (H) *PEDV* S protein‐specific IgG/IgA levels by ELISA. (I–K) Cellular immune profiles of PBLs isolated from sows at farrowing. (I) Flow cytometric quantification of the proportions of CD3^+^CD4^+^ and CD3^+^CD8^+^ T cells stained with fluorophore‐conjugated anti‐porcine CD3, CD4, and CD8 monoclonal antibodies. (J, K) Secretion of cytokines. PBLs were stimulated with recombinant *PEDV* S protein for 24 h, and the concentrations of (J) IL‐4 and (K) IFN‐γ in cell supernatants were quantified using ELISA kits. All data are expressed as the mean ± standard deviation (SD), *n* = 3 sows per experimental group. Statistical comparisons were performed using one‐way analysis of variance (ANOVA) with Tukey’s multiple comparison post‐hoc test. The asterisks indicate significant differences between groups, with *p* < 0.05 ( ^∗^ *p* < 0.05;  ^∗∗^ *p* < 0.01; ns, not significant).

The immunization schedule for pregnant sows is shown in Figure 1A. Dynamic changes in serum neutralizing antibody titers of pregnant sows over a 120‐day period following the primary immunization are presented in Figure 1B–D. Among the three immunized groups, the serum neutralizing antibody titers against the GⅡb genotype *PEDV* were ranked, descending order: MV + MV group > AV + IV group > IV + IV group (Figure 1B). All three immunization regimens maintained robust neutralizing antibody titers against the GⅡb genotype for up to 30 days postfarrowing, thereby providing a basis for passive immune protection in neonatal piglets. Notably, sera from animals immunized with GⅡb *PEDV* strain displayed weak cross‐neutralization activity against GI and GⅡa *PEDV* genotypes, as shown in Figure 1C,D. This finding aligns with prior studies on antigenic divergence between *PEDV* genotypes. Furthermore, the trend in neutralizing antibody titer detected in sow colostrum collected on the day of farrowing was consistent with that observed in serum (Figure 1G), suggesting efficient transfer of maternally derived neutralizing antibodies from sows to neonatal piglets via colostrum.

### 3.2. *PEDV S*‐Specific IgG and IgA Kinetics in Gestating Sows Detected by ELISA

Kinetics of serum *PEDV*–specific IgG and IgA levels in gestating sows across 120 DPP are presented in Figure 1E,F. Across the three vaccinated groups, serum IgG levels targeting the *PEDV* S protein exhibited the descending order: IV + IV group > MV + MV group > AV + IV group. All three immunization regimens maintained robust IgG antibody concentrations for up to 30 days postfarrowing, matching the neutralizing antibody trends and further verifying effective maternally derived antibody transmission. In contrast, the serum IgA concentrations exhibited a distinct ranking: the AV + IV group > MV + MV group > IV + IV group. Both AV + IV and MV + MV regimens sustained relatively high IgA concentrations within 30 days postfarrowing, whereas the IV + IV group consistently retained weak IgA titers across the entire sampling window. These data indicate that prime‐boost protocols including AV are superior in eliciting IgA responses, which are central to mucosal defense against enteric coronavirus infection. Consistent with serum antibody profiles, colostrum *PEDV* S protein‐specific IgG and IgA concentrations harvested at farrowing closely matched serum levels (Figure 1H). This finding confirms efficient transfer of maternally derived IgG and IgA into colostrum, thereby providing dual humoral immune protection to neonatal piglets through colostrum ingestion.

### 3.3. Lymphocyte Subset Proportions and Cytokine (IL‐4/IFN‐γ) Production in Sows at Farrowing

The proportions of PBL subsets in sows at farrowing are shown in Figure 1I. Relative to the control group, the AV + IV and MV + MV vaccination regimens induced significant elevations in the proportions of CD3^+^CD4^+^ and CD3^+^CD8^+^ T cells (*p* < 0.05 and *p* < 0.01, respectively), demonstrating their efficacy in eliciting robust cellular immune responses. In contrast, the IV + IV group exhibited no significant differences in CD3^+^CD4^+^ or CD3^+^CD8^+^ T cells compared with the control group (*p* ≥ 0.05), suggesting that the IV‐only regimen induced relatively weak cellular immunity. These findings were further validated by the IFN‐γ date (Figure 1K). Following stimulation with the *PEDV* S protein, PBLs from the AV + IV and MV + MV groups secreted significantly higher concentrations of IFN‐γ than those from the IV + IV group and control group (*p* < 0.05). This outcome aligns with the enhanced T cell subset responses observed in these groups as IFN‐γ is a key Th1–type cytokine mediating antiviral cellular immunity. In contrast, IL‐4 production exhibited a different trend (Figure 1J). PBLs from all three immunized groups produced elevated levels of IL‐4 after *PEDV* S protein stimulation, with the IV + IV group showing the highest IL‐4 concentration (*p* < 0.05 compared with the other two immunized groups). IL‐4 is a major Th2 cytokine that drives B‐cell proliferation and antibody secretion. Combined with the high IgG (Figure 1E) and moderate IgA (Figure 1F) observed in the IV + IV group, it confirms that the IV‐only regimen preferentially induces humoral immune responses.

### 3.4. Dynamic Changes of Passive Immunity Antibodies in Piglets Induced by Different Maternal Immunization Regimens

**Figure 2:**
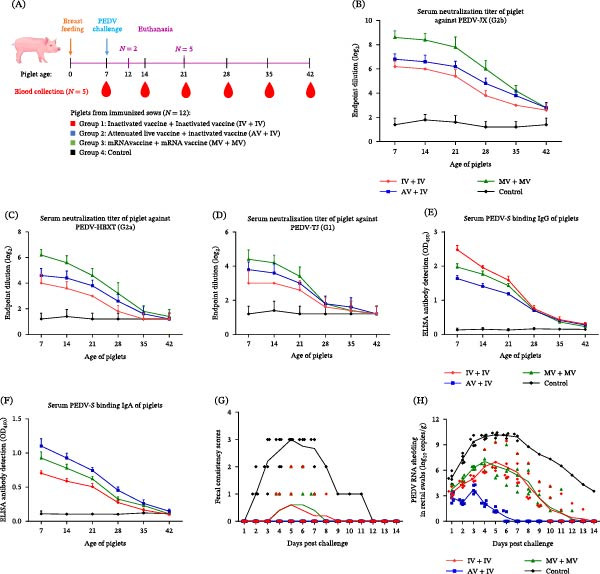
Passive immune antibody dynamics, challenge protection efficacy, and viral shedding in piglets derived from immunized sows. (A) Experimental design for piglet passive immunity and *PEDV* challenge. Piglets were born to sows assigned to four immunization regimens (Groups 1–4): Group 1 (IV + IV), inactivated vaccine + inactivated vaccine; Group 2 (AV + IV), attenuated live vaccine + inactivated vaccine; Group 3 (MV + MV), mRNA vaccine + mRNA vaccine; Group 4 (negative control), PBS + PBS (0.01 M, pH 7.2). Groups 1–3 served as passive immune protection groups and Group 4 as the negative control. (B–F) Passive immune antibody dynamics: Five piglets per group were randomly selected, and blood samples were collected at 7, 14, 21, 28, 35, and 42 days of age to detect. (B) Neutralizing antibody titers against genotype GⅡb *PEDV*; (C) cross‐neutralizing antibody titers against genotype GⅡa *PEDV*; (D) cross‐neutralizing antibody titers against genotype GⅠ *PEDV*; (E) *PEDV* S protein‐specific IgG levels measured via ELISA; (F) *PEDV* S protein‐specific IgA levels measured via ELISA. (G, H) Evaluation of protective efficacy and viral shedding following *PEDV* challenge. Ten piglets from each group were randomly selected and challenged via orogastric gavage with PEDV JX strain (P5) at 7 days of age. Five piglets per group were euthanized and necropsied at 5 dpc and the remaining five at 14 dpc. (G) Fecal consistency was scored daily postchallenge according to the standardized scale to assess diarrhea incidence. (H) Rectal swabs were collected daily postchallenge, and *PEDV* RNA loads were quantified by qRT‐PCR to evaluate viral shedding dynamics.

As shown in Figure 2A, blood samples were collected from piglets at 7, 14, 21, 28, 35, and 42 days of age to measure neutralizing antibody titers and *PEDV* S protein‐specific IgG and IgA levels (Figure 2B–F). The overall kinetics of passive antibody responses in piglets, including neutralizing antibodies, IgG, and IgA, closely mirrored those observed in lactating sows, indicating efficient transfer of maternal antibodies via colostrum and milk. Piglets maintained high neutralizing antibodies against genotype GⅡb *PEDV* up to 21 days of age, followed by a rapid decline thereafter (Figure 2B). This decrease coincided with weaning, suggesting that the cessation of maternal antibody intake through milk is a major contributing factor. Consistent with the maternal serum profiles, sera from piglets exhibited weak cross‐neutralizing activity against GⅡa genotype *PEDV* strain (Figure 2C) and GⅠ genotype *PEDV* strain (Figure 2D), further confirming the limited cross‐protective efficacy among *PEDV* genotypes. The IgG and IgA in piglets (Figure 2E,F) also decreased sharply after 21 days of age, paralleling the neutralizing antibody kinetics and reflecting the physiological waning of maternally derived passive immunity. Notably, distinct passive antibody profiles were observed among the three immunization groups. Piglets in the MV + MV group exhibited the highest neutralizing antibody titers, accompanied by moderate levels of IgG and IgA. Piglets in the AV + IV group showed intermediate neutralizing antibody titers but the highest IgA concentrations, along with relatively low IgG levels. In contrast, piglets in the IV + IV group displayed the lowest neutralizing antibody titers, characterized by the highest IgG levels and comparatively low IgA levels. Taken together, these results indirectly support a positive association between neutralizing antibody titers and IgA concentrations [27]. Although IgG may contribute to enhancing neutralizing activity, it appears insufficient to fully compensate for IgA in mediating protective immunity against *PEDV*, particularly given the intestinal tropism of the virus and the critical role of mucosal IgA in blocking viral invasion.

### 3.5. Diarrhea and Viral Shedding in Piglets With Passive Immunity After PEDV Challenge

As depicted in Figure 2A, piglets were challenged with the *PEDV* JX strain via an oral gavage at 7 days of age. Rectal swabs were collected daily for 14 consecutive dpc to evaluate fecal consistency and quantify *PEDV* RNA levels, with the corresponding results shown in Figure 2G,H, respectively. Fecal consistency was scored according to the standardized scoring system described in Section 2.8. Notably, deaths or severe diarrhea in piglets (score = 3) were not observed in any of the three passively immunized groups. Mild diarrhea (score = 2) occurred in one piglet each in the IV + IV and MV + MV groups, whereas no piglets in the AV + IV group exhibited diarrhea (score = 0). In contrast, piglets in the control group developed severe diarrhea as early as 3 dpc, followed by progressive mortality, with only one piglet surviving throughout the 14 days. Analysis of *PEDV* RNA levels in rectal swabs revealed distinct viral shedding patterns among the groups. Piglets in the AV + IV group exhibited the lowest viral RNA levels, which were significantly reduced compared with the other groups (*p* < 0.05), and viral RNA became undetectable by 7 dpc, indicating efficient viral clearance. In the IV + IV and MV + MV groups, viral RNA levels were lower than those in the control group (*p* < 0.05) but higher than those in the AV + IV group (*p* < 0.05). In the IV + IV and MV + MV groups, viral RNA levels decreased sharply by 7 dpc; however, viral RNA became undetectable by 14 dpc only in the MV + MV group, while shedding persisted in the IV + IV group beyond this time point. Piglets in the control group exhibited sustained high viral RNA levels from 3 to 10 dpc, with four out of five succumbing to diarrhea‐induced dehydration between 4 and 8 dpc and the sole surviving piglet continuing to shed *PEDV* for more than 14 days.

### 3.6. Clinical Necropsy and Histopathological Examination of Piglets Post‐PEDV Challenge

**Figure 3:**
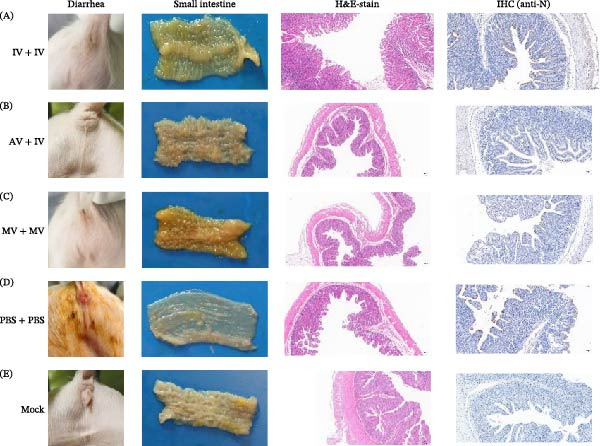
Clinical signs, necropsy findings and histopathological examination of piglets post‐*PEDV* challenge. (A–E) Diarrhea status, gross pathological lesions of the small intestine, and histopathological examinations (H.E staining and IHC) at 5 dpc, samples were collected from neonatal piglets born to gestating sows immunized with IV + IV, AV + IV, MV + MV, or PBS control regimens, all of which received *PEDV* oral challenge at 7 days of age as well as mock‐infected piglets without PEDV challenge.

At 5 dpc, two nondiarrheic piglets were selected per immunization group (IV + IV, AV + IV, and MV + MV) for euthanasia and necropsy (diarrheic individuals excluded to minimize interference with clinical observations). Conversely, two diarrheic piglets were randomly chosen from the challenge control group. Jejunal tissues were collected for histopathological analysis (Figure 2A). Viral shedding profiles (Figure 2H) confirmed successful *PEDV* infection in all piglets, and the severity of diarrhea was positively correlated with viral load (Figure 2G). At 5 dpc, piglets born to sows immunized with the IV + IV regimen exhibited discernible intestinal architecture. H&E staining revealed mild injury and exfoliation of the intestinal villous epithelium; *PEDV* antigens were detected in villous epithelial cells by IHC (Figure 3A). In contrast, piglets from the AV + IV group displayed a well–preserved intestinal morphology and intact villous epithelium, with no viral antigens detected via IHC (Figure 3B), indicating effective passive immune protection. Piglets in the MV + MV group showed gross and microscopic pathological changes similar to those in the IV + IV group, including slight villous epithelial damage and positive viral signals in epithelial cells (Figure 3C). As expected, piglets in the PBS control group developed severe diarrhea, accompanied by a disrupted intestinal structure, severe villous epithelial injury, shedding, and widespread distribution of viral antigens (Figure 3D). No obvious pathological lesions were observed in normal control piglets (Figure 3E). Collectively, all three maternal vaccination regimens conferred partial passive immune protection to suckling piglets compared with the blank control group. Among them, the AV + IV regimen provided relatively superior protective efficacy, whereas the IV + IV and MV + MV regimens exhibited comparable protection, only partially mitigated *PEDV* infection, and reduced intestinal tissue injury.

## 4. Discussion

*PEDV* continues to impose a significant burden on the swine industry, particularly affecting neonatal piglets during lactation. In China, mortality rates in neonatal piglets ranged from 82% to 95% in 2024, resulting in direct annual economic losses exceeding 4.7 billion CNY [28]. *PEDV* is transmitted primarily via the fecal–oral route and aerosolized particles and exhibits marked environmental stability, remaining infectious for up to 175 days at −20°C [29]. Rapid viral evolution has led to genotype mismatches between currently available vaccines and circulating field strains [30]. Moreover, outdated immunization protocols and insufficient induction of colostral IgA responses further compromise vaccine efficacy [31]. Collectively, these factors facilitate recurrent *PEDV* outbreaks and underscore the challenges of achieving sustained disease control. Passive immunity is critical for protecting neonatal piglets, whose adaptive immune system remains immature at birth. Maternal antibodies are transferred exclusively through colostrum within the first 24 h postfarrowing, with IgG accounting for approximately 85% of total colostral immunoglobulins. Effective Fc receptor‐mediated intestinal absorption requires the ingestion of at least 150 mL of colostrum within the first 6 h after birth [32]. Notably, sows naturally exposed to field *PEDV* strains develop elevated colostral IgA levels, which play an essential role in limiting intestinal viral invasion, consistent with the enteric tropism of *PEDV* [33]. These findings highlight the importance of inducing robust maternal IgA responses to achieve effective passive immune protection for neonatal piglets.

The immunization regimens evaluated in this study, intramuscular IV followed by intramuscular IV (IV + IV), AV followed by IV (AV + IV), and modified MV followed by modified MV (MV + MV), were selected based on clinically mainstream protocols and advanced research models. Specifically, IV + IV represents the conventional regimen used in large‐scale farms [34]; AV + IV is the preferred heterologous prime‐boost strategy in farms with high *PEDV*incidence [18]; MV + MV reflects a homologous boost design for MVs [17, 35]. All immunizations were administered at commercially approved and clinically recommended doses, with the primary objective of screening practical regimens and verifying real‐world vaccine efficacy under routine clinical conditions, rather than comparing antigen dosage across vaccine types. Thus, this study aimed to provide practical data to inform clinical vaccine applications. Notably, the AV + IV heterologous prime‐boost regimen conferred markedly effective passive immune protection in piglets, as evidenced by significantly reduced clinical symptoms (e.g., diarrhea incidence), lower viral shedding, and alleviated intestinal pathological lesions compared with the IV + IV and MV + MV groups. Although the number of piglets subjected to necropsy (two per group) and the overall sample size were limited, the consistent superiority of the AV + IV group in serum antibody titers, cellular immune responses, and key protective indicators (e.g., survival rate and intestinal integrity) collectively supports its advantage as a practical immunization strategy for clinical application. This finding aligns with previous reports that heterologous combinations of live attenuated and IVs enhance lactogenic immunity and improve passive protection in offspring [36]. Interestingly, the MV + MV group exhibited higher neutralizing antibody levels but relatively poorer protective efficacy. This discrepancy may be largely attributable to weaker immune memory responses induced by MVs. Although MVs elicited the highest acute neutralizing antibody titers, they showed rapid antibody decay and inferior quality of memory B cells, characterized by limited affinity maturation and breadth [37, 38], which failed to sustain sufficient and effective protective immunity against the *PEDV* challenge. Moreover, the immune efficacy of MVs is closely associated with the compatibility of cationic lipids in the delivery system and the rational design of mRNA sequences [39]. The two‐dose IV regimen (IV + IV) showed the weakest protective effect, primarily due to its failure to induce robust *PEDV*–specific IgA production. Inactivated vaccination does not effectively polarize CD4^+^ T helper cells toward the Th1 phenotype, leading to insufficient secretion of key cytokines such as IFN‐γ. Additionally, the activation of CD8^+^ cytotoxic T cells is impaired, which largely explains its poor immune‐protective efficacy [40]. In contrast, primary immunization with an AV recruits dendritic cells, T cells, and IgA^+^ B cells, thereby establishing a foundation for IgA production. Subsequent booster immunization with an IV further amplifies humoral and cellular immune responses, significantly increasing specific IgA levels [36]. This further corroborates that IgA serves as a critical protective antibody capable of blocking *PEDV* invasion into the intestinal mucosa [41].

Nevertheless, this study has an inherent limitation in its experimental design. The absence of an AV + AV immunization control group precludes the delineation of the independent contributions of AV priming versus IV boosting to the enhanced immune efficacy observed with the AV + IV regimen. Future studies incorporate an AV + AV control group to clarify the respective immunological roles of different vaccine platforms and further optimize rational prime‐boost vaccination strategies against *PEDV* infection. The *PEDV* challenge dose used in this study was selected based on the physiological characteristics of 7‐day‐old early‐weaned piglets fed with a milk replacer under laboratory conditions. This experimental model was established to evaluate passive immune protection in suckling piglets derived from immunized sows. The applied challenge dose stably induced typical diarrhea symptoms and 80% mortality in control piglets, which was sufficient to distinguish immune protective efficacy among different vaccination groups. Given the high stress susceptibility of early‐weaned piglets, an excessively high challenge dose (10‐ or 100‐fold higher) would cause universal severe infection and near‐complete mortality across all groups, rendering it impossible to effectively differentiate vaccine efficacy. Therefore, the current moderate challenge dose is reasonable and feasible for this passive immunity evaluation model under laboratory conditions. However, this challenge dose is considerably lower than the natural infection dose encountered under clinical field conditions and thus cannot fully reflect vaccine preventive effects in real‐world scenarios. This may introduce a risk of false‐positive protective results, implying that the actual protective efficacy under field conditions would be inferior to that verified in the laboratory. It should also be noted that this study focused solely on serum antibody profiles and clinical phenotypes (e.g., diarrhea incidence, mortality, viral shedding), lacking mechanistic validation at the intestinal level‐specifically, the detection of intestinal cytokines (e.g., IL‐4, IL‐17, and IFN‐γ) and lymphocyte subsets (e.g., IgA^+^ B cells and T helper subsets). Future investigations are warranted to incorporate these mechanistic assays to elucidate the regulatory pathways underlying IgA production and cellular immune activation, thereby providing more robust theoretical support for optimizing *PEDV* immunization strategies.

In conclusion, this study systematically evaluated the passive immune‐protective efficacy of three conventional sow immunization regimens against *PEDV*. The results demonstrated that sequential prime‐boost immunization with an AV followed by an IV (AV + IV) induced robust *PEDV*–specific humoral and cellular immune responses in sows, thereby providing reliable and durable passive protection for neonatal piglets when the vaccine strain matched the prevalent field genotype. In contrast, the homologous MV regimen (MV + MV) elicited high levels of acute neutralizing antibodies but exhibited insufficient immune memory persistence and suboptimal cellular immune induction, resulting in limited overall protective efficacy. Although further optimization of mRNA sequence design and lipid delivery systems is required, MVs remain a promising candidate for future *PEDV* vaccine development. Collectively, these findings clarify the immune superiority of the attenuated‐inactivated combined immunization strategy, provide a valuable experimental basis for the rational selection and optimization of clinical sow immunization protocols, and offer theoretical references for the subsequent improvement and translational application of MVs against swine enteric coronavirus diseases.

## Author Contributions

**Chen Sun**: conceptualization (assisted), data curation, formal analysis (figure/table analysis), writing – original draft. **Juan Jin and Li Zhao**: validation (data verification), writing – review and editing (draft revision). **Meng Hao**: writing – review and editing (draft revision). **Maojie Lv**: validation (data verification). **Endong Bao**: project administration, data management, validation (data verification), writing – review and editing, supervision.

## Funding

This study was supported by Ringpu Biotechnology Co., Ltd., Tianjin, China (Project Number ZXYF0034).

## Disclosure

All authors have read and approved the final manuscript. The funder had no role in study design, data collection and analysis, decision to publish, or preparation of the manuscript.

## Ethics Statement

All animal procedures were performed in compliance with the ARRIVE guidelines and the 3R principles. The study protocol was approved by the Institutional Animal Care and Use Committee (IACUC) of Ringpu Biotechnology Co., Ltd. (Approval Number IACUC–AX2505R0–024). Animals were housed under standard laboratory conditions with free access to food and water, and all efforts were made to minimize suffering.

## Conflicts of Interest

The authors declare no conflicts of interest.

## Data Availability

The data that support the findings of this study will be deposited in a publicly accessible repository with a permanent DOI upon formal acceptance of this manuscript for publication. Data are available from the corresponding author upon reasonable request in the interim.
